# Carotid Arterial Stiffness and Hemodynamic Responses to Acute Cycling Intervention at Different Times during 12-Week Supervised Exercise Training Period

**DOI:** 10.1155/2018/2907548

**Published:** 2018-07-16

**Authors:** Hai-Bin Liu, Wen-Xue Yuan, Qing-Yong Wang, Yan-Xia Wang, Hou-Wen Cao, Jian Xu, Kai-Rong Qin

**Affiliations:** ^1^Department of Physical Education, Dalian University of Technology, Dalian 116024, China; ^2^School of Biomedical Engineering, Faculty of Electronic Information and Electrical Engineering, Dalian University of Technology, Dalian 116024, China; ^3^Department of Engineering Mechanics, Dalian University of Technology, Dalian 116024, China; ^4^School of Optoelectronic Engineering and Instrumentation Science, Dalian University of Technology, Dalian, 116024, China

## Abstract

This paper studied the alterations in arterial stiffness and hemodynamic responses during resting state and immediately following acute cycling intervention at different times across 12-week supervised exercise training. Twenty-six sedentary young males participated in the exercise training program at moderate intensity. Arterial stiffness and hemodynamic variables of the right common carotid artery were measured and computed during resting state and immediately following acute cycling intervention at weeks 0, 4, 8, and 12. Across the 12-week exercise training, carotid arterial stiffness was decreased at weeks 8 and 12 and hemodynamic variables were improved at week 12 during resting state. In response to acute cycling intervention, carotid arterial stiffness exhibited an acute increase foremost at 8 weeks, and arterial maximal and mean diameters showed acute decreases at weeks 0 and 4. Despite significant differences in arterial stiffness and hemodynamic variables between resting state and immediately after acute intervention for each time period, these differences presented a progressive decrease across the 12-week exercise training. In conclusion, long-term exercise training not only improved carotid arterial stiffness and hemodynamic alterations when at rest but also negated the acute responses of carotid arterial stiffness and hemodynamic variables to acute cycling intervention.

## 1. Introduction

Common carotid arteries are the main conduits that supply blood to the brain. The changes in the structure and function of common carotid arteries are relevant to the occurrence and development of atherosclerosis, coronary ischemia, and stroke [1]. Carotid arterial stiffness is an independent predictor of these cardio- and cerebrovascular events [2, 3]. Hemodynamic variables, including blood pressure, blood flow-induced wall shear stress (WSS), and oscillatory shear index (OSI) not only play crucial roles in mediating arterial stiffness but also are considered as important biomechanical markers of the carotid arterial structures and functions [4].

Exercise training can modulate arterial stiffness and directly induce systemic and local arterial hemodynamic responses [5]. Cross-sectional comparisons between sedentary individuals and athletes, including runners, cyclists, and basketball players [6–8], have demonstrated that arterial stiffness and hemodynamic parameters, such as arterial diameter, blood supply to the brain, and blood pressure have been improved. Longitudinal investigations regarding the effects of exercise training on sedentary individuals with cardiovascular disease have also shown that acute and long-term supervised exercise training change arterial stiffness and arterial hemodynamics [9].

It has been reported that increased arterial stiffness is observed after acute resistance training [10] and high-intensity dynamic cycling [11], whereas a decrease or no change in arterial stiffness is detected following a bout of aerobic exercise of low to moderate intensity [12, 13]. Therefore, there is a significant relationship between exercise intensity of long-term aerobic training and cardiovascular improvement [10, 14, 15]. Extensive investigations [7–9, 16–18] have demonstrated that arterial stiffness and hemodynamics in well-trained athletes exhibit no obvious changes to acute intervention, while those in matched control groups are significantly altered in response to acute intervention. In addition, some studies have documented that 4 weeks [19] and 8 weeks of cycling [20] as well as 6 weeks of resistance training [21] improve the arterial function and hemodynamic responses. Aforementioned disparate findings may be due to exercise modality, the differences in subjects, exercise duration time, or the time points of experimental measurement.

Therefore, to better evaluate the effects of exercise training on arterial stiffness and hemodynamics, the same types of exercise modality and the same experimental subjects need to be chosen in one study, and intensive measurement time points should be set during long-term exercise training. Furthermore, due to the fact that the majority of investigations [22, 23] mentioned above only focused on the effects of acute exercise intervention alone or on the long-term exercise training [9, 24], so the effects of arterial stiffness and hemodynamic response to acute cycling exercise at different times point during a period of exercise training are unclear, which need to be further elucidated.

In order to solve the above problems, 26 young healthy sedentary men were recruited, and four weekly measurements across a 12-week period of supervised exercise training were conducted. The stiffness and local hemodynamic forces of the right common carotid artery were obtained at rest and immediately after acute cycling exercise using the same workload for each lab visit. The present study will supply hemodynamic indicators induced by exercise training for mediating the arterial function and structure, furthermore achieving a better understanding of the arterial function and hemodynamic response to acute exercise interventions with the progression of exercise training.

## 2. Methods

### 2.1. Ethical Approval

Ethical approval was obtained from the Ethics Committee of Dalian University of Technology, and the study was carried out in accordance with the Declaration of Helsinki (1964). Informed consent was acquired from the subjects prior to the onset of acute cycling intervention and exercise training.

### 2.2. Subjects

A total of 32 young male subjects were recruited from the community, and they consented to participate in the present study. The entry criteria included being a nonsmoker and having sedentary individuals who were not involved in any regular planned exercise program [25]. None of the subjects reported having been diagnosed with cardiovascular disease, diabetes, respiratory problems, or any musculoskeletal disorders. Six of the subjects withdrew owing to a lack of interest in the study during the 12-week training period. Twenty-six subjects (age, 22.54 ± 2.56 years; stature, 1.75± 0.06 m; mass, 70.36 ± 6.54 kg; BMI, 22.53 ± 1.62 kgm^−2^) completed the 12-week exercise training period, including acute cycling intervention and hemodynamic measurements.

### 2.3. Study Design

The subjects visited the lab four times during the supervised exercise training during weeks 0, 4, 8, and 12. Subjects reported to the laboratory at the same time of day, 3 hours after a meal. For each measurement visit, subjects were asked to refrain from caffeine and exercise before each visit. During each visit, vascular ultrasound assessments were conducted after the subjects lay in a supine position for more than 15 min. Subsequently, the subjects performed an acute leg cycling exercise after a 5-minute warm-up of cycling. Finally, they resumed the supine position for an immediate vascular ultrasound measurement.

### 2.4. Acute Cycling Intervention and Exercise Training

**Figure 1:**
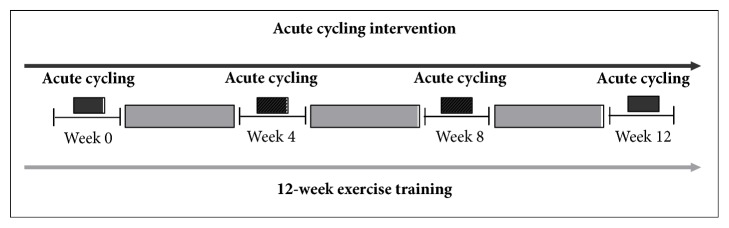
Schematic presentation of acute cycling intervention and exercise training.

A schematic presentation of acute cycling intervention and exercise training is provided in Figure 1. The acute cycling exercise was conducted once for 30 s on a bicycle ergometer (Powermax-VIII, Combi Wellness, Japan) at weeks 0, 4, 8, and 12 during the exercise training period. The workload was set at 250 W and cadence was maintained at 60–70 RPM.

The supervised exercise training consisted of 60 min sessions three times a week during the 12-week period on a running track. Each session included a 10 min warm-up and 5 min of stretching, followed by 30 min of running at a moderate intensity of 60 to 70% of heart rate reserve (American College of Sports Medicine-ACSM, 2000), a 10 min cool down period, and 5 min additional stretching. During the supervised exercise training, the subjects wore a heart rate monitor (Geonaute OR310, Decathlon, France).

### 2.5. Vascular Ultrasound Measurement

The subjects lay in a supine position for ≥15 minutes. Images of the right common carotid artery were obtained using Doppler ultrasound (ProSound Alpha 7, Aloka) and 7.5-10.0 MHz linear-array probe. Images were acquired 5-10 mm below the carotid bulb. The common carotid artery was assessed using a longitudinal view. The distance from the near wall to far wall lumen-intima interface was continuously traced using eTracking software and used to determine carotid artery diameters. Center-line velocity waveforms were measured using range gated spectral Doppler signals averaged along the Doppler beam. An insonation angle ⩽ 60° was maintained for all measures and sample volume was manually adjusted to encompass the entire vessel. Synchronously, a cuff-type manometer (Patient Monitor PM8000, Mindray) positioned on the upper-left arm was used to record the heart rate, brachial systolic pressure (*p*_*s*_*mea*_), and diastolic pressure (*p*_*d*_*mea*_). Eventually, the same vascular ultrasound assessment procedure was performed immediately following the acute cycling exercise. All heart rate and blood pressure measurements were registered in triplet, and the average of three values was adopted for subsequent analysis.

### 2.6. Carotid Vascular Measurement

**Figure 2:**
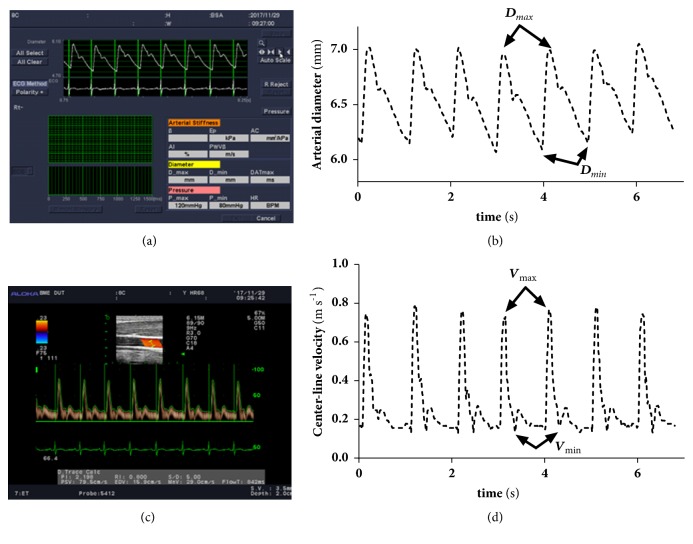
Images and extractions of arterial diameter and center-line velocity waveforms.

The Aloka in-built software (using eTrack wall-tracking) detected center-line velocity (Figure 2(a)) and arterial diameter waveform (Figure 2(c)) to save only as images; therefore self-compiled program in Matlab was used to extract blood vessel diameter (Figure 2(b)) and center-line velocity waveforms (Figure 2(d)). Electrocardio signals were used to synchronize diameter and center-line waveforms. *D*_*max*_, *D*_*min*_, and *D*_*mean*_ are the maximal, minimal, and mean arterial diameters. *V*_*max*_, *V*_*min*_, and *V*_*mean*_ are the maximal, minimal, and mean center-line velocities.

### 2.7. Hemodynamic Computations

Hemodynamic parameters were computed using (1)–(11) below, in which the blood viscosity* η *or blood density *ρ* was determined as the same values for all subjects, i.e., *η = *0.004 Pa·s and *ρ* = 1050 kg/m^3^, and the maximal harmonic number *n*= 20 was chosen for (3), (4), and (8).

### 2.8. Calibration of Carotid Blood Pressure

**Figure 3:**
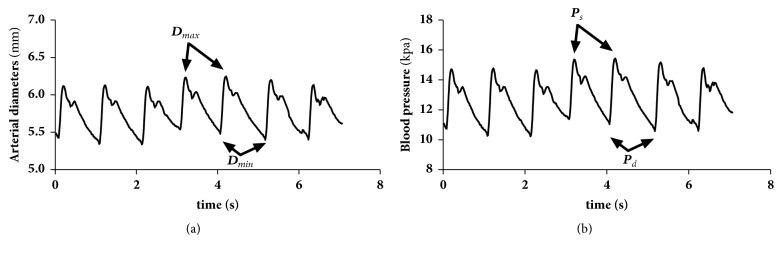
Arterial diameter and carotid blood pressure waveforms.

There was a linear relationship between the changes in vessel diameter and the changes in blood pressure [26] within each cardiac cycle (the coefficient of fit is above 0.97). Therefore, the waveforms of blood pressure and the diameter in the carotid artery are similar (see Figure 3). In this study, we considered the mean value of the carotid arterial pressure *p*_*m*_ and diastolic pressure *p*_*d*_ to be approximately equal to the mean value of the brachial pressure *p*_*m*_*mea*_ and diastolic pressure *p*_*d*_*mea*_, as done in the literature [27]. The mean carotid arterial pressure (*p*_*m*_) was calculated using the following approximate equation:

(1) $$\begin{array}{c} p_{m}=p_{m\_mea}=p_{d\_mea}+\frac{1}{3}\left(\;p_{s\_mea}-p_{d\_mea}\;\right) \end{array}$$

Therefore, the carotid artery blood pressure waveform was calibrated using the brachial mean arterial* p*_*m*_ and diastolic pressure* p*_*d *_(*p*_*d_mea*_). The maximal value of the carotid artery waveform was calculated and assumed to be the systolic pressure* p*_*s*_.

### 2.9. Flow Rate* (FR)*

The FR was calculated for

(2) $$\begin{array}{c} Q=2\pi {R_{0}}^{2}\overset{1}{\underset{0}{\int }}y\cdot u\left(\;y\;\right)dy \end{array}$$

where* R*_0_ is the time-averaged value of the carotid artery radius during one cardiac cycle, *y* = *r*/*R*_0_, in which* r* is the radial coordinate, and *u*(*y*) meets [28]

(3) uy,t=∑n=−∞+∞J0αnj3/2−J0αnj3/2yJ0αnj3/2−1u0,ωnejωnt

where* n *is the harmonic number,* J*_*0 *_is the 0^th^-order Bessel function of the first kind, $j=\sqrt{-1}$, $\alpha_{n}=R_{0}\sqrt{\rho \omega_{n}/\eta }$ is the Womersley number,* ρ *is the density of blood,* η *is the blood viscosity, *ω*_*n*_ = 2*nπf* is the angular frequency,* f *is the base frequency, and *u*(0, *ω*_*n*_) is the* n *harmonic component of the measured center-line velocities and satisfies

(4) $$\begin{array}{c} u\left(\;0,t\;\right)=\overset{+\infty }{\underset{n=-\infty }{\sum }}u\left(\;0,\omega_{n}\;\right)e^{j\omega_{n}t} \end{array}$$

In addition, *Q*_*max*_, *Q*_*min*_, and *Q*_*mean*_ are the maximal, minimal, and mean blood flow FR during one cardiac cycle.

### 2.10. Pressure-Strain Elastic Modulus (*E*_*p*_)

The pressure-strain elastic modulus (*E*_*p*_) or Peterson modulus [29], which is a measure of blood vessel elasticity, was applied using the following equation:

(5) $$\begin{array}{c} E_{p}=\frac{p_{s}-p_{d}}{R_{s}-R_{d}}\cdot R_{d} \end{array}$$

### 2.11. *β*-Stiffness Index (*β*)

As a means of adjusting the arterial compliance for changes in distending pressure, *β* was calculated as follows [30]:

(6) $$\begin{array}{c} \beta =\frac{\ln\left(p_{s}/p_{d}\right)}{R_{s}-R_{d}}\cdot R_{d} \end{array}$$

### 2.12. Circumferential Strain (CS)

CS was determined through the following equation [31]:

(7) $$\begin{array}{c} CS=\frac{R_{s}-R_{d}}{R_{d}} \end{array}$$

### 2.13. Wall Shear Stress (WSS)

WSS (*τ*_*w*_), which is the frictional force of blood flow acting upon the vascular endothelium [32], was determined using [28]

(8) $$\begin{array}{c} \tau_{w}=\frac{\eta }{R_{0}}\overset{+\infty }{\underset{n=-\infty }{\sum }}\frac{\alpha_{n}j^{3/2}J_{1}\left(\alpha_{n}j^{3/2}\right)}{J_{0}\left(\alpha_{n}j^{3/2}\right)-1}u\left(\;0,\omega_{n}\;\right)e^{j\omega_{n}t} \end{array}$$

where *J*_1_ is a first-order Bessel function of the first kind, and *τ*_*w*_*max*_, *τ*_*w*_*min*_, and *τ*_*w*_*mean*_ are the maximal, minimal, and mean WSS waveforms during a cardiac period.

### 2.14. Oscillatory Shear Index (OSI)

The* OSI* index represents the WSS acting in directions other than the direction of the temporal mean WSS vector and was provided by Ku et al. [33].

(9) $$\begin{array}{c} OSI=\frac{1}{2}\left(\;1-\frac{\left|\int_{0}^{T}\tau_{w}dt\right|}{\int_{0}^{T}\left|\tau_{w}\right|dt}\;\right) \end{array}$$

where *T* is the period of each cardiac cycle.

### 2.15. Peripheral Resistance (*PR*)

*PR*, resistance to the flow of blood, is determined by the vascular musculature and diameter of the blood vessels and is responsible for the blood pressure when coupled with the flow rate as follows:

(10) $$\begin{array}{c} PR=\frac{P_{mean}}{Q_{mean}} \end{array}$$

### 2.16. Dynamic Resistance (*DR*)

*DR* is defined as the relationship between changes in blood pressure and alterations in blood flow rate as

(11) $$\begin{array}{c} DR=\frac{P_{s}-P_{d}}{Q_{max}-Q_{min}} \end{array}$$

### 2.17. Statistical Analysis

All data are presented as the mean (SD) and statistical significance was assumed to be* P*< 0.05. Data are presented for an at-rest state (●) and postcycling (□). The repeated ANOVA was used to assess the differences between the baseline and weeks 4, 8, and 12 during the 12-week exercise training period. An ANCOVA with a baseline score as a covariate was used for a statistical analysis to compare the differences between at-rest and postcycling intervention for each lab visit. Statistical analyses were conducted using SPSS 19.0 (SPSS Inc., Chicago, IL, USA) software.

## 3. Results

### 3.1. Arterial Stiffness and Diameter Responses at Different Times during the Training Period

**Figure 4:**
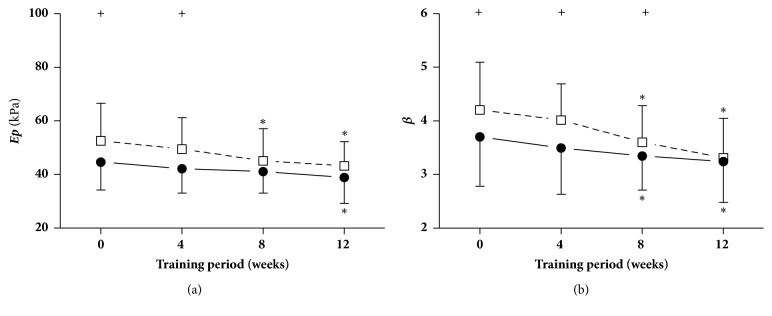
**Pressure-strain elastic modulus (** **E** **p** **) and arterial stiffness index (** **β** **)**. Data are presented for at rest (●) as well as after cycling (□). + Significant difference between at rest and after cycling:* P* < 0.05. *∗* Significant difference from baseline within group:* P* < 0.05.

Figure 4 shows the pressure-strain elastic modulus (*Ep*) and arterial stiffness index (*β*) when at rest and immediately following cycling intervention at different points in time, i.e., weeks 0, 4, 8, and 12. It can clearly be seen from Figure 4 that during the 12-week exercise training period,* Ep* and *β* were significantly decreased at weeks 8 and 12, respectively, compared with week 0. In response to acute cycling intervention,* Ep *increased significantly at weeks 0 and 4, whereas *β* significantly increased at weeks 0, 4, and 8 in comparison with those during an at-rest state.

**Figure 5:**
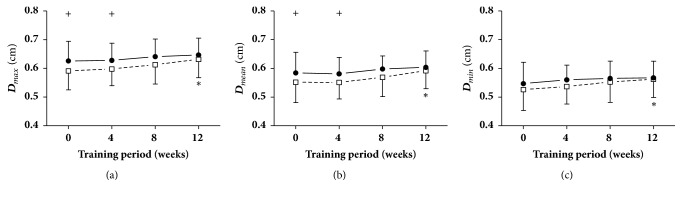
**Arterial diameters (D)**. Data are presented for at rest (●) as well as after cycling (□). + Significant difference between at rest and after cycling:* P* < 0.05. *∗* Significant difference from baseline within group:* P* < 0.05.

The maximal, mean, and minimal diameters at rest and immediately following cycling intervention at different points in time (weeks 0, 4, 8, and 12) are shown in Figure 5. It is clear that during the 12-week exercise training period, the maximal, mean, and minimal diameters immediately after acute cycling were significantly increased at week 12 compared with week 0. In addition, the maximal and mean diameters immediately after exercise at weeks 0 and 4 were remarkably decreased in comparison to those during an at-rest state.

### 3.2. Carotid Blood Flow Responses at Different Times during the Course

Table 1 lists the changes in carotid blood flow at different time periods during the exercise training course. During the 12-week exercise training period, compared to week 0, the HR at week 12 during an at-rest state and the HR at weeks 8 and 12 immediately after acute cycling were clearly decreased; in addition, ***V***_***mean***_ at week 12 and **Q**_*min*_ at weeks 4, 8, and 12 immediately after exercise, and **Q**_*max*_ and **Q**_*mean*_ when at rest at week 12, were remarkably increased. In response to acute cycling intervention, the HR, ***V***_*max*_, ***V***_*mean*_, ***V***_*min*_, ***Q***_*max*_, and ***Q***_*mean*_ increased significantly at different time periods, with the exception of***Q***_*mean*_ at week 12, whereas ***Q***_*min*_ decreased significantly at the four measurement times.

### 3.3. Hemodynamic Responses at Different Times during the Training Period

**Figure 6:**
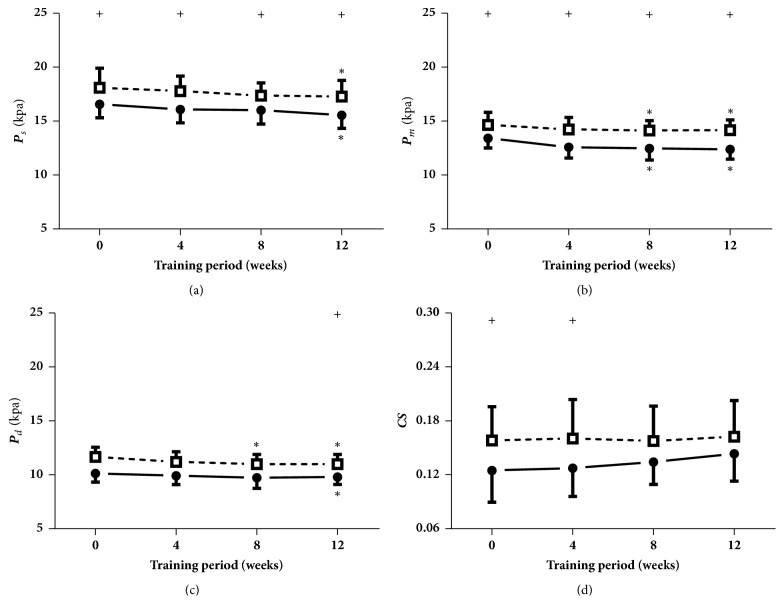
**Blood pressure (P) and circumferential strains (CS)**. Data are presented for at rest (●) as well as after cycling (□). + Significant difference between at rest and after cycling:* P* < 0.05. *∗* Significant difference from baseline within group:* P* < 0.05.

As indicated in Figure 6, the mean pressure during both states and the diastolic pressure immediately after cycling at week 8, as well as the systolic, diastolic, and mean pressure during both states at week 12, were significantly reduced compared with those at week 0. After the interventions of acute cycling, the systolic and diastolic pressure at the four measured times, the diastolic pressure at week 12, and the circumferential strains at weeks 0 and 4 were remarkably elevated.

**Figure 7:**
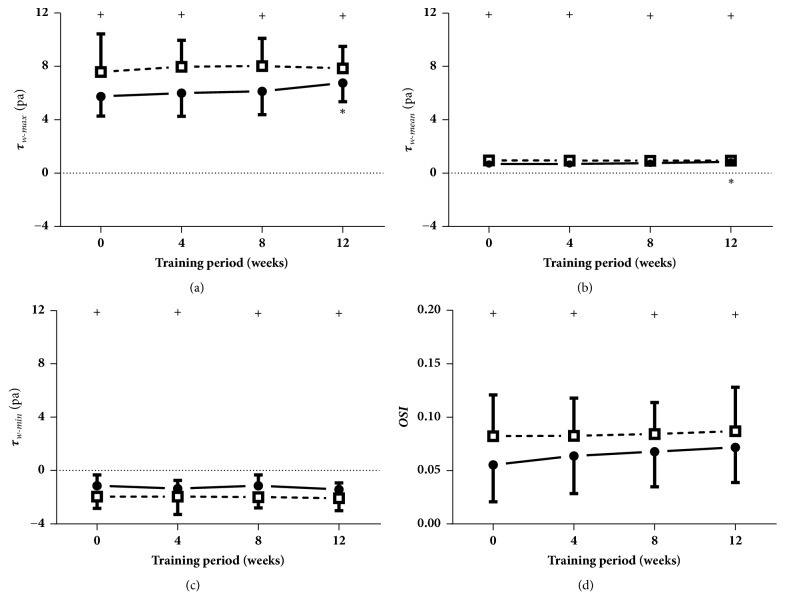
**Wall shear stress (WSS) and oscillatory shear indexes (OSI)**. Data are presented for at rest (●) as well as after cycling (□). + Significant difference between at rest and after cycling: P < 0.05. *∗* Significant difference from baseline within group: P < 0.05.

As shown in Figure 7, compared to week 0, the maximal WSS and mean WSS when at rest were significantly increased at week 12. Additionally, the maximal WSS and mean WSS and OSI were remarkably increased, and the minimal WSS was clearly decreased after the acute cycling exercise at the four measurement times.

**Figure 8:**
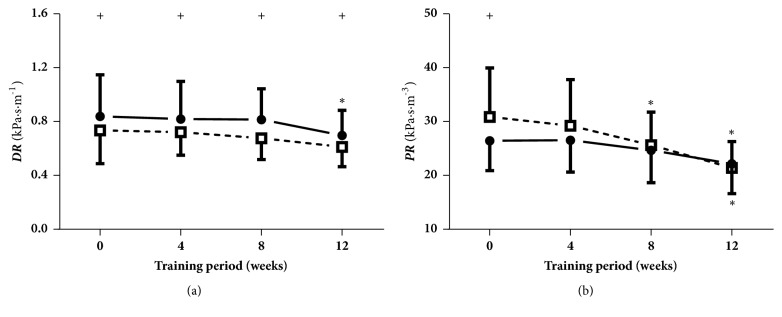
**Dynamic resistance (DR) and peripheral resistance (PR)**. Data are presented for at rest (●) as well as after cycling (□). + Significant difference between at rest and after cycling:* P* < 0.05. *∗* Significant difference from baseline within group:* P* < 0.05.

Figure 8 demonstrates the dynamic resistance (DR) and peripheral resistance (PR) at rest and immediately following cycling intervention at four measured times (weeks 0, 4, 8, and 12). As can be seen, the PR immediately after the acute cycling exercise at week 8 and the DR at rest and the PR during both states at week 12 were clearly reduced in contrast with those at week 0. In addition, the DR at the four measured times was remarkably decreased, whereas the PR at week 0 was significantly increased, as induced through the acute cycling exercise.

## 4. Discussion

Acute exercise intervention is an effective method for investigating the cardiovascular functional response to external stimuli, whereas repeated episodic bouts of long-term exercise training induce chronic functional adaptation, and ultimately structural arterial remodeling [9]. However, the majority of previous studies have been concerned with only the effects of acute exercise intervention or long-term exercise training. This is the first study to examine not only the alteration in arterial stiffness and hemodynamics when at rest but also arterial stiffness and hemodynamic responses after acute cycling intervention at 4-week intervals across a 12-week exercise training period at moderate intensity* in vivo*. The main results regarding arterial stiffness and hemodynamics at rest during the 12-week exercise training period can be summarized as follows. (1) The carotid arterial stiffness was decreased at week 8, whereas the arterial diameter was not changed at week 8 or 12 when at rest. (2) Carotid blood flow was improved at week 12, whereas a decrease in heart rate occurred at week 12. (3) Systolic pressure, diastolic pressure, dynamic resistance, and peripheral resistance were decreased, whereas the maximal WSS and mean WSS were increased, at week 12. In addition, arterial stiffness and hemodynamic responses to acute cycling intervention across the 12-week exercise training program are as follows. (1) Arterial stiffness was increased and the diameters were decreased the most at week 8 of exercise training, with no significant changes in arterial stiffness and diameters shown in week 12. (2) Despite the significant differences in carotid blood flow and the hemodynamic forces (BP, CS, WSS, OSI, DR, and PR) between periods of at-rest and postcycling intervention for each time period, a progressive decrease was presented across the 12-week exercise training period.

Regular aerobic-endurance exercise, such as running or cycling, has a positive effect on arterial stiffness [34], whereas different time durations of exercise training, such as 4 [19] and 8 weeks [20], have a different impacts on arterial stiffness. Decreased carotid arterial stiffness in this study was detected at week 8. A meta-analysis demonstrated that the effect of aerobic-endurance exercise training on arterial stiffness was enhanced with higher exercise intensity [10]. In addition, several cross studies [8, 35] have indicated that endurance-trained athletes have larger arteries than the control subjects. Longitudinal studies have also reported significant increases in the dimensions of the ascending and abdominal aorta, as well as the femoral artery in a trained limb, but not in an untrained limb after 6 weeks of one-legged cycle exercise [36]. In addition, enhanced femoral artery diameters were observed after 3 months of walking exercise [37]. However, the 12 weeks of moderate-intensity exercise training in this study did not induce a significant increase in the carotid arterial diameters. Tinken's group [18] examined the time period of vascular adaptation of the conduit artery function and vasodilator capacity while at rest during an 8-week exercise training period. The differences in the above-mentioned results regarding arterial stiffness and diameters may have occurred from the use of different subjects, exercise intensities, or intervention protocols applied. Moreover, long-term aerobic-endurance exercise has been well accepted as incurring a larger blood supply to the brain, as well as an improved heart rate and blood pressure [38]. The results of the present study have further confirmed these investigations.

On the other hand, hemodynamic alterations induced through exercise training have been confirmed to play a role in cardiovascular disease reduction [39]. The cardioprotective effect of exercise training may be mediated by improvements in endothelial function [40], with the proposed mechanism involving repeated increases in WSS [41]. Cell cultures, animal, and human studies have demonstrated that the increased WSS upregulates the endothelial nitric oxide synthase activity in the blood vessels [40, 42]. It has been reported that the carotid blood flow and WSS decrease with age in healthy individuals [43]. This finding has been confirmed by other studies [44]. Although hemodynamic force, namely, WSS, has been accepted as a crucial determinant of arterial function and vascular remodeling, no additional information is available concerning the impact of regular exercise training on the WSS during a particular period of time. In the present study, the increased maximal and mean WSS and blood flow were significantly increased at week 12. Therefore, this research demonstrated that long-term exercise training has a significant impact on an improvement in WSS, which may further lead to arterial remodeling, such as an increase in arterial diameter.

Acute exercise intervention induces immediate changes in artery function and structure. A novel aspect of the current study is detecting arterial stiffness and hemodynamic responses to acute cycling interventions at different times across the 12-week supervised exercise training period. Considerable research efforts [45] have demonstrated that the effects of acute exercise intervention in central stiffness are dependent on the intensity of exercise. Continuous acute exercise of moderate intensity and accumulative exercise over time have both been shown to reduce arterial stiffness [46], whereas acute high-intensity exercise has resulted in decrease in common carotid artery dimensions and an increase in stiffness [11]. It should be acknowledged that exercise intensity is a relative concept for different subjects, from beginners to experienced athletes, as well as individuals at different time points during an exercise training session. To gain a better understanding of how arterial stiffness and hemodynamic responses differ at various points in time during exercise training, the same intensity of cycling intervention was adopted in this study. At 8 weeks of exercise training, arterial stiffness increased, and the diameters were decreased the most, whereas arterial stiffness and diameters were not changed at week 12. Consequently, acute cycling intervention was a relatively high-intensity stress for the subjects who performed the exercise training during the first 8-week period, whereas the same intensity stimuli had no significant impact for them with the remaining weeks of the exercise training.

Beneficial alterations have been observed following aerobic exercise training, whereas potentially detrimental effects occur with high-intensity acute exercise training. An acute bout of high-intensity cycling was shown to result in a significant increase in fluctuations of blood pressure and blood flow [11]. High flow organs, for example, the brain and eyes, are particularly sensitive to extra pressure and flow pulsatility, which facilitate penetration of exaggerated pulsatile energy into the microvascular bed [47]. This may lead to repeated episodes of microvascular ischemia, and tissue damage. In addition, these extracranial hemodynamic responses induced by acute exercise may exert detrimental effects on the carotid arteries and cerebrovascular circulation [11]. A comparison of the at-rest state and immediately after cycling intervention for each period of time measured in the present study indicates that blood supply to brain and hemodynamic forces (BP, CS, WSS, OSI, DR, and PR) presented a progressive decrease across the training period. Taken together, this research extends our knowledge that long-term exercise training improves arterial stiffness when at rest, particularly negating the hemodynamic responses to acute cycling intervention for sedentary populations through progressive long-term exercise training. Moreover, these findings indicate that the arterial function and hemodynamic responses to acute exercise intervention follow a particular time frame. Exercise training at moderate intensity improved the arterial function and hemodynamics at weeks 8 and 12, respectively. Recently, high-intensity interval training has become increasingly popular among young individuals. The results from previous studies [11, 47] as well as the present research demonstrate that acute high-intensity exercise intervention may have a detrimental effect on the carotid arterial function and structure, particularly the brain for exercise beginners. The findings in this study imply that long-term exercise training with moderate intensity not only improves the arterial hemodynamics when at rest but also enhances the arterial adaptation to counteract the negative effects of these extra cranial hemodynamic responses induced through acute exercise. Furthermore, this paper suggests that sedentary people who wish to undertake high-intensity exercise training may need to first undergo a period of moderate-intensity exercise training of 8 to 12 weeks to decrease the detrimental effects of high-intensity training on their carotid arteries and cerebrovascular circulation.

## 5. Limitations

There are some limitations in the present study worth addressing. First, participant recruitment may be misleading for including only males and not including females. Literature [48, 49] and results in our group [50] demonstrated that there were significant differences between males and females in arterial stiffness and hemodynamic variables at rest or after acute exercise interventions. Cyclic changes in sex hormones in women may affect carotid stiffness [51] although this is not a universal finding [11]. Future research is needed to explore possible acute and chronic effects of long-term exercise and acute intervention on arterial stiffness and hemodynamic response in females. Second, in this study, we took arterial stiffness and hemodynamic responses of our volunteers at rest state as the control. We did not recruit a separate group of subjects and use their arterial stiffness and hemodynamic response as the control. Third, carotid pressure was not measured directly from the common carotid region. We used the brachial mean and diastolic blood pressure to calibrate the carotid waveform and computed the carotid systolic blood pressure. Further research will be carried out to achieve a better understanding of the arterial function and hemodynamic response to acute exercise interventions with the progression of exercise training through controlling the limitations.

## 6. Conclusion

In the present study, a 12-week exercise training period at moderate intensity decreased arterial stiffness and altered the hemodynamic forces at different periods of time. In response to acute exercise intervention, arterial stiffness increased and the diameters decreased the most at week 8 of the exercise training, whereas arterial stiffness and diameters showed no significant responses at week 12. In addition, the hemodynamic responses presented a progressive decrease across the 12-week exercise training period. In summary, long-term exercise not only improves arterial stiffness and hemodynamic alterations at rest state but also negates the detrimental arterial stiffness and hemodynamic responses to acute exercise intervention.

## Figures and Tables

**Table 1:** The changes in carotid blood flow at rest and after acute cycling across 12-week training.

**Variable**	**Condition**	**Week 0**		**Week 4**		**Week 8**		**Week 12**	
**HR**	At rest	72±7		71±7		69±6		67±6*∗*	
	Acute cycling	97±10	+	96±9	+	91±11*∗*	+	89±10*∗*	+
***V***_*max*_	At rest	68±11		71±12		72±11		73±10	
	Acute cycling	92±22	+	94±18	+	94±17	+	95±13	+
***V***_*mean*_	At rest	23±5		24±5		25±6		26±4	
	Acute cycling	29±7	+	30±7	+	31±5	+	33±5*∗*	+
***V***_*min*_	At rest	11±4		13±5		14±5		14±3	
	Acute cycling	15±6	+	16±7	+	17±6	+	18±8	+
***Q***_*max*_	At rest	11.64±1.78		12.02±2.17		13.76±2.29		14.49±2.41*∗*	
	Acute cycling	16.82±4.17	+	16.97±4.57	+	17.09±6.28	+	17.32±4.84	+
***Q***_*mean*_	At rest	3.22±0.53		3.36±0.67		3.51±0.69		4.08±0.78*∗*	
	Acute cycling	4.40±0.89	+	4.44±1.43	+	4.34±1.35	+	4.38±1.16	
***Q***_*min*_	At rest	0.56±1.64		0.62±2.05		0.63±1.38		0.75±1.49	
	Acute cycling	-1.65±1.03	+	-0.94±1.55*∗*	+	-1.01±1.58*∗*	+	-1.07±2.25*∗*	+

*∗*: significant difference from baseline at *P*<0.05; +: significance between at rest and after acute cycling at *P*<0.05; **HR**, heart rate (beats/min); ***V***_*max*_, ***V***_*mean*_, and ***V***_min_, maximal, mean, and minimal center-line velocity (**CV**, cm/s); ***Q***_***max***_, ***Q***_***mean***_, and ***Q***_***min***_, maximal, mean, and minimal flow rate (**FR**, ml/s).

## Data Availability

The data used to support the findings of this study are available from the corresponding author upon request.
